# Prism adaptation does not alter configural processing of faces

**DOI:** 10.12688/f1000research.2-215.v1

**Published:** 2013-10-14

**Authors:** Janet H. Bultitude, Paul E. Downing, Robert D. Rafal

**Affiliations:** 1 Oxford Centre for Functional Magnetic Resonance Imaging of the Brain (FMRIB), Nuffield Department of Clinical Neurosciences, University of Oxford, Oxford, OX3 9DU, UK; 2 Wolfson Centre for Clinical and Cognitive Neuroscience, School of Psychology, Bangor University, Gwynedd, LL57 2AS, UK

## Abstract

Patients with hemispatial neglect (‘neglect’) following a brain lesion show difficulty responding or orienting to objects and events on the left side of space. Substantial evidence supports the use of a sensorimotor training technique called prism adaptation as a treatment for neglect. Reaching for visual targets viewed through prismatic lenses that induce a rightward shift in the visual image results in a leftward recalibration of reaching movements that is accompanied by a reduction of symptoms in patients with neglect. The understanding of prism adaptation has also been advanced through studies of healthy participants, in whom adaptation to leftward prismatic shifts results in temporary neglect-like performance. Interestingly, prism adaptation can also alter aspects of non-lateralised spatial attention. We previously demonstrated that prism adaptation alters the extent to which neglect patients and healthy participants process local features versus global configurations of visual stimuli. Since deficits in non-lateralised spatial attention are thought to contribute to the severity of neglect symptoms, it is possible that the effect of prism adaptation on these deficits contributes to its efficacy. This study examines the pervasiveness of the effects of prism adaptation on perception by examining the effect of prism adaptation on configural face processing using a composite face task. The composite face task is a persuasive demonstration of the automatic global-level processing of faces: the top and bottom halves of two familiar faces form a seemingly new, unknown face when viewed together. Participants identified the top or bottom halves of composite faces before and after prism adaptation. Sensorimotor adaptation was confirmed by significant pointing aftereffect, however there was no significant change in the extent to which the irrelevant face half interfered with processing. The results support the proposal that the therapeutic effects of prism adaptation are limited to dorsal stream processing.

## Introduction

Patients with hemispatial neglect (‘neglect’) following a brain lesion show difficulty responding or orienting to objects and events that appear on the left side of space ^1^. A diagnosis of neglect is a strong predictor of poor functional outcome and low independence following stroke ^2^. This may be partly because the disorder impairs perception in a broad range of sensory modalities ranging from vision, touch, proprioception, and motor control to more abstract aspects of cognition such as a patient’s awareness of their own body ^3^ and their imagined images of familiar locations ^4^. Furthermore, although the rightward spatial bias is the defining symptom of neglect, several other processing disturbances are associated with the disorder. These include low general arousal ^5^, poor sustained attention ^6^, and difficulties in keeping track of spatial locations as they move about their environment ^7^. These non-lateralised spatial biases are thought to increase neglect severity and reduce the potential for recovery ^8^.

Over the last fifteen years a promising behavioural intervention for neglect has emerged in the form of a sensorimotor training technique called prism adaptation ^9^. During prism adaptation, patients reach for objects viewed through rightward-deflecting prisms, leading to a leftward recalibration of reaching movements that can be measured as leftward errors once the prisms are removed. In patients with neglect this leftward recalibration of reaching is accompanied by a reduction in their symptoms. A single five-minute session of prism adaptation is sufficient to improve the performance of neglect patients on tests of visuo-motor function such as copying, cancellation and reading ^9, 10^. These effects extend to non-visual spatial processing, such as tactile perception ^11^ and manual exploration of space while blindfolded ^12^, and to complex mental operations such as the exploration of an internally generated map of France ^13^; and 'bisection' of numbers ^14^. Evidence amassed over a number of studies suggests that this simple behavioural intervention can have broadly generalised effects, and prism adaptation is considered to be a highly promising potential treatment for neglect ^15^.

Whereas adaptation to rightward-shifting prisms can reduce neglect symptoms in brain-lesioned patients, adaptation to leftward-shifting prisms, involving a rightward recalibration of reaching, leads to neglect-like changes in the spatial performance of healthy participants. These perceptual changes have been demonstrated on a similar range of visual, non-visual and mental tasks (albeit to a lesser extent than those changes observed in patients) ^17– 19^. Since prism adaptation can be used to induce similar, but opposite, changes in the performance of healthy participants as in neglect patients, it is possible to gain insights into the potential therapeutic effects of the technique by testing healthy volunteers.

One example of research from healthy participants that has complemented the understanding gained from studies in patients is in research examining the effects of prism adaptation on non-lateralised deficits. There are now several pieces of evidence from brain-lesioned patients that prism adaptation alters spatial processing deficits that cannot be described in terms of orienting to the left versus the right, including reductions in spatial dysgraphia ^20^ and shifts ^21^ and reductions in perseveration ^22^. We previously demonstrated that adaptation to rightward-shifting prisms reverses the tendency of patients with right hemisphere lesions to become fixated on local details of a scene in preference to the global configuration (the ‘local processing bias’) ^23^. Patients identified the local or global level of large letters that were built from smaller letters (‘Navon’ figures). Reaction times to the local level increased after prism adaptation, demonstrating that there was a reduction in patients’ ability to identify the local level without interference from conflicting information at the global level. Conversely, RTs to the global level decreased following prism adaptation, demonstrating that patients were better able to ignore irrelevant conflicting information from the local level. In a similar experiment with healthy participants we demonstrated that adaptation to leftward-shifting prisms temporarily increased local processing ^24^, and led to neglect-like errors in the way in which a spatial representation or ‘map’ of the environment is updated as we move our gaze around it ^25^. Together these results demonstrate that prism adaptation has a more pervasive influence on visual perception than merely shifting attention to one side.

**Figure 1.**
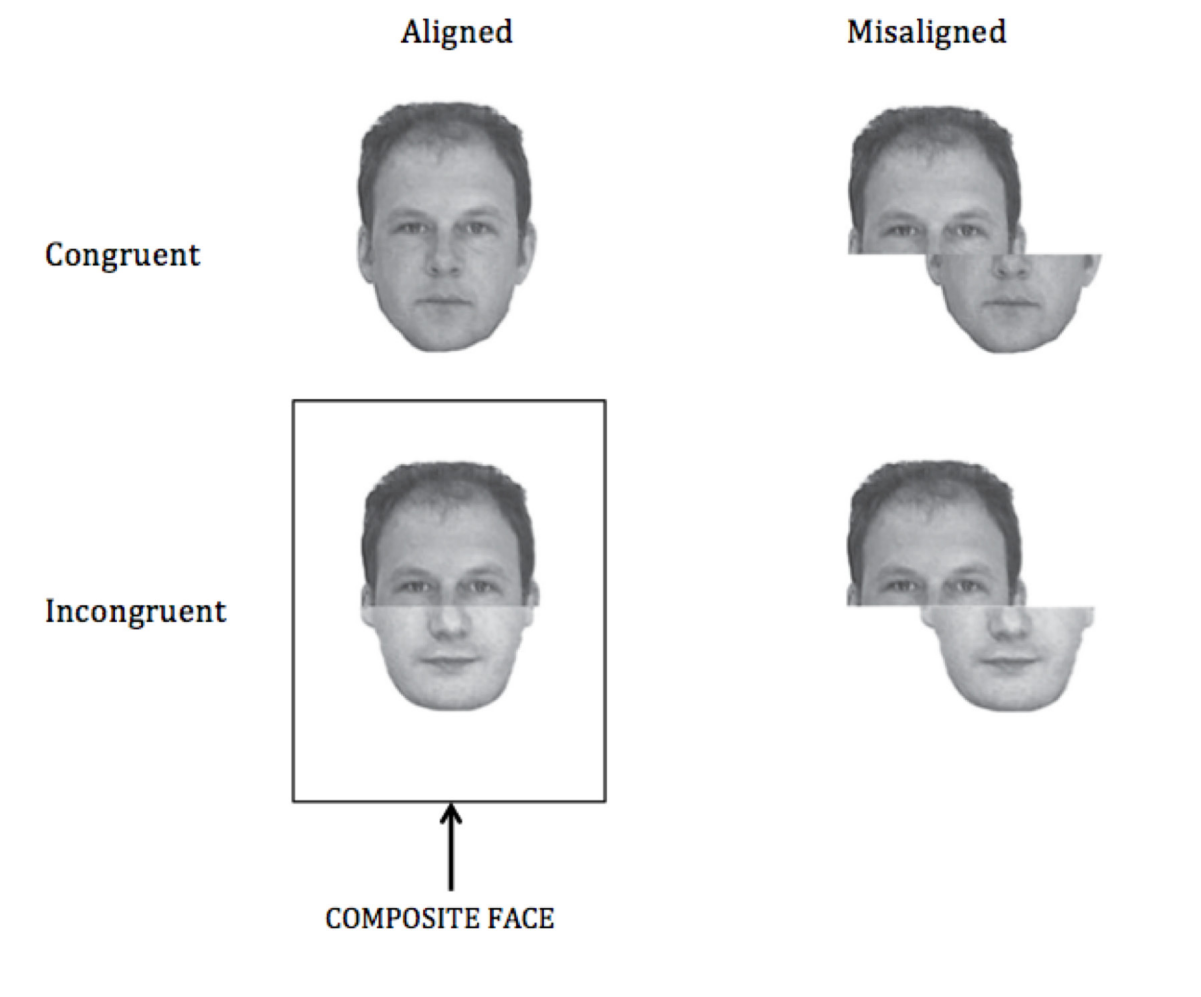
Examples of the four stimulus types (adapted from Weston and Perfect ^30^). Stimuli for the task used in the present study took the same form as these examples, but were created from black-and-white publicity photographs of two well-known movie stars (Brad Pitt and George Clooney).

To further test the extent of this influence, the present study examines the effect of prism adaptation on the perception of composite faces in healthy participants. Faces, perhaps more than any other object, undergo automatic global-level processing in which individual components are highly integrated and less available to independent evaluation. This is powerfully illustrated in the composite face illusion ( Figure 1): when the upper and lower halves of two faces are recombined, the virtually unavoidable illusion is that one is viewing the face of a third, different person. When participants are asked to identify the top or bottom halves of composites that are formed from faces of well-known celebrities, they are slower compared to when performing the same task when the two face halves are offset ^26^. This reaction time cost demonstrates that even when processing a face as an integrated Gestalt would impair our ability to perform the task at hand, we are unable to suppress such configural processing.

We had two main reasons for testing the influence of prism adaptation on configural face perception. First, by using a stimulus type for which normal processing is known to be strongly biased towards global processing, we reasoned that we could gain insight into the pervasiveness of the influence of prism adaptation on perceptual processes. Second, this experiment explores the possibility that prism adaptation could be used to improve face processing in individuals with prosopagnosia and autism, who have been shown to have reduced or absent configural face processing ^27, 28^. We predicted that adaptation to leftward-shifting prisms, which induced neglect-like processing in healthy participants, would reduce the RT cost associated with identifying composite faces. We further predicted that there would be no change in composite face processing following adaptation to rightward-shifting prisms, which does not induce perceptual changes in healthy participants.

## Material and methods

Sixty-four right-handed undergraduate women (mean age=19.8 years, *SEM*=0.32; mean handedness=-0.83, *SEM*=0.026 where a score of -1 denotes complete right-handedness; ^29^) completed a composite face task before and after a brief (five-minute) session of prism adaptation (see below for a full description of the task). Only female participants were selected for the study as it was felt that the stimuli - images of Brad Pitt and George Clooney - might have, on average, higher saliency for women than men. To be included in the study participants were also required to have normal or corrected-to-normal vision, and full use of their right arm. Informed consent was obtained in accordance with guidelines approved by the Bangor University ethics committee and the 2008 Declaration of Helsinki. Participants received course credits for the 45-minute session.

In a repeated-measures design, participants completed one set of configural face processing tasks before prism adaptation, and one set of configural face processing tasks after prism adaptation.

### Prism adaptation and open-loop pointing

Prism adaptation and confirmation of sensorimotor realignment were performed using a similar procedure as that used for prism adaptation treatment of hemispatial neglect ^24^. For prism adaptation, participants made 150 visually-guided pointing movements while wearing goggles fitted with prismatic lenses that shifted the visual field 15° to the left or right. In order to confirm adaptation, a participant pointed under target lines while vision of their pointing arm was occluded by a panel ('open-loop pointing'). Twelve open-loop pointing trials were performed immediately before and after prism adaptation (‘pre-' and ‘post-test’). In order to confirm that the sensorimotor realignment was retained throughout the entire post-adaptation configural face processing task, a third set of open-loop pointing errors were recorded at the end of the experiment (‘late-test’). Open-loop pointing error was measured by the experimenter to the nearest 0.5°, with negative numbers indicating leftward errors and positive numbers indicating rightward errors.

Participants performed a composite face task using stimuli similar to those used by Weston and Perfect ^30^. Figure 1 (adapted from Weston and Perfect ^30^) provides examples of the four stimulus types used in the present experiment. Stimuli for the composite faces task had the same form as these examples, but were created from black-and-white publicity photographs of two well-known movie stars (Brad Pitt and George Clooney). All stimuli were constructed from the same two images and were presented on a black background. All participants correctly named the celebrities when shown these photographs at the beginning of the experimental session. Congruent stimuli were the unaltered pictures: that is the top and bottom face-halves were from the same celebrity. Incongruent stimuli were constructed by combining top and bottom face-halves from the two different celebrities. Faces were presented with the top and bottom halves aligned, or with the top half of the face offset to the left or right with reference to the bottom half, by approximately one-third of the face half. Participants identified the top or bottom half of each face in separate, identical blocks. For each trial, a fixation cross appeared for 500 ms, followed by the face stimulus for 200 ms, then a blank screen. Responses were made by pressing one of two buttons on a keyboard with the index or middle finger of their right hand. The participant’s response ended the trial. Each block consisted of 32 repetitions of each of the four stimulus types (congruent-aligned, congruent-misaligned, incongruent-aligned and incongruent-misaligned) in pseudorandom order, resulting in a total of 128 trials per block. Block order (top first or bottom first) and key allocation (Brad-left-George-right or George-left-Brad-right) were counterbalanced between participants.

Statistical analyses were performed using SPSS software ^31^. Pointing errors and reaction time (RT) data were subjected to repeated-measures ANOVAs. Follow-up paired-t-tests were performed using Bonferroni correction for multiple comparisons.

## Results

### Open-loop pointing

Data File 1 contains the full pointing data for each participant. A mixed ANOVA of pointing errors with the factors Prism Group (leftward, rightward) and Session (pre, post, late) revealed a significant two-way interaction [ *F*(2,124)=213.2, *p*<0.001]. This reflected a significant rightward shift in pointing error for the leftward-shifting prism group between the pre-test ( *M*=-0.2, *SEM*=0.33) and the post-test [ *M*=4.9, *SEM*=0.28; *t*(31)=15.0, *p*<0.001], which was still significant in the late-test [ *M*=3.6, *SEM*=0.27; *t*(31)=10.4, *p*<0.001]. Similarly, there was a significant leftward shift in for the rightward-shifting prism group between the pre-test ( *M*=0.7, *SEM*=0.35) and the post-test [ *M*=-3.3, *SEM*=0.40; *t*(31)=12.1, *p*<0.001], and this was sustained to the late-test [ *M*=-2.7, *SEM*=0.39; *t*(32)=8.7, *p*<0.001]. Comparison of 95% Confidence Intervals around the pre- to post-test pointing shifts indicated that there were no significant differences in the absolute magnitude of the after-effect for the two groups. Similarly, there were no significant differences in the pre- to late-test pointing shifts. Therefore prism adaptation resulted in significant shifts in open-loop pointing error in both groups, which were maintained for the entire duration of the post-adaptation composite face task.

Data File 1 (spreadsheet 1): Mean pre-, post- and late-test pointing errors for all participants. Values are in degrees of visual angle, with negative numbers indicating a leftward error, and positive values indicating a rightward error.

### Composite face task

Mean accuracy was at ceiling (93%), precluding meaningful analysis. For each participant, responses that were faster than 200 ms or more than 3 SD above their mean RTs were excluded from analysis. Four participants demonstrated low accuracy for incongruent trials (>3 SD from mean error rate) during one or more block of the experiment, suggesting a failure to comprehend or comply with task instructions (i.e., their responses suggested that these participants were identifying, for example, the top half of the faces in a block in which they had been instructed to identify the bottom half of the faces). These participants were excluded from the analyses. Data for one of the experimental blocks was missing for two participants due to an error made by the experimenter. Since the responses of these individuals were otherwise similar to the remaining participants (suggesting that they were able to understand the instructions) these participants were retained and their missing data was replaced by the mean for that group.

For each prism group (leftward- or rightward-prisms), repeated-measures ANOVAs were conducted on the RT cost of alignment; that is, the difference between RTs for aligned and misaligned faces. By this index, a larger RT cost indicates greater interference due to configural processing, and a small RT cost indicates that participants were able to focus on the face halves with little or no interference from configural processing. The key factors of interest for the analyses were Prism (pre, post) and Congruency (congruent, incongruent). Previous studies have demonstrated temporal limitations to the effects of Navon figure processing on changes in the recognition of pre-learned faces ^32^ and composite halves ^30^, with the effects decaying by the second half of the post-induction test phase. In order to test for such changes over time, we therefore included two further time-based factors in our analyses: Block Number (first, second) and Block Half (first, second). Finally, since any time-based effects may also be influenced by which half of the face participants identified immediately after prism adaptation, a between-subjects factor of Block Order (top-half-first, bottom-half-first) was also included. Therefore, the ANOVAs included five factors: Prism, Congruency, Block Order, Block Number and Block Half.

The full data for the analysis are presented in Data File 2. The analyses revealed significant main effects of Congruency for both leftward-shifting [M=-6.5 vs M=16.1; *F*(1,28)=28.6, *p*<0.001] and rightward-shifting [M=0.15 vs M=14.1; *F*(1,29)=913.0, *p*<0.005] prism groups, reflecting lower RT costs of alignment for the incongruent faces than for the congruent faces. A significant main effect of Block Half for the rightward-shifting prism group indicated higher RT costs for trials in the first half of the block compared to the second half [M=12.7 vs M=-1.6; *F*(1,29)=5.5, *p*<0.05]. A significant Block Order x Block Number interaction for the leftward-shifting prism group [ *F*(1,27)=4.78, *p*<0.05] reflected trends in the RT cost of alignment depending on which face half the participants identified. That is, there was a non-significant tendency towards a higher RT cost of alignment for block two for participants in the ‘bottom half first’ group, and for block one for the participants in the ‘top half first’ group ( *p*s>0.05).

Data File 2 (spreadsheets 2-5): RT costs of alignment for each condition for the composite face task. RT costs were calculated by subtracting the raw RT for the misaligned condition from the RT for the aligned condition. FH=First half of the experimental block; SH=Second half of the experimental block; I=incongruent, C=congruent.
Spreadsheet 2 = Pre-adaptation, First experimental block; Spreadsheet 3 = Pre-adaptation, Second experimental block; Spreadsheet 4 = Post-adaptation, First experimental block; Spreadsheet 5 = Post-adaptation, Second experimental block

**Figure 2.**
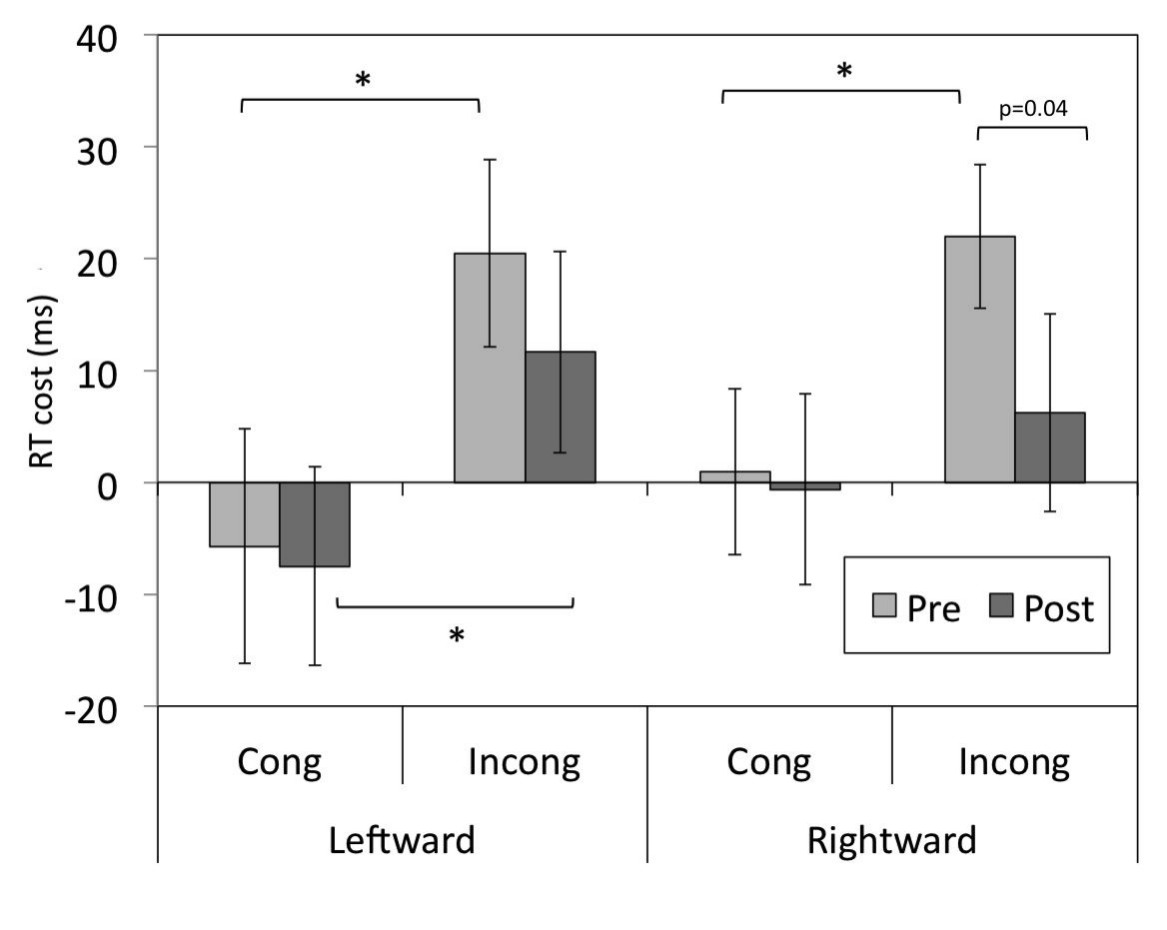
Data for the Prism x Congruency interaction. RT costs of alignment for congruent and incongruent trials before and after adaptation to leftward- and rightward-shifting prisms. Error bars represent ± SEM, *=significant to a Bonferroni-corrected alpha level of *p*=0.0125.

There was no significant interaction of Prism and Congruency for the leftward-shifting prism group ( *p*s>0.05), although a trend for a Prism x Congruency interaction arose for the rightward-shifting prism group [ *F*(1,29)=3.4, *p*=0.074]. The RT costs for this interaction are plotted in Figure 2 for both groups, and follow-up t-tests were performed on an *a priori* basis. In contradiction of the experimental hypothesis, there was no significant change in RT cost of alignment for congruent or incongruent faces following adaptation to leftward-shifting prisms. There was, however, a trend for a reduction in RT cost for incongruent faces for participants in the rightward-shifting prism (control) group [ *t*(30)=2.2, *p*=0.04, assessed to a Bonferroni-corrected alpha-level of *p*=0.0125].

**Figure 3.**
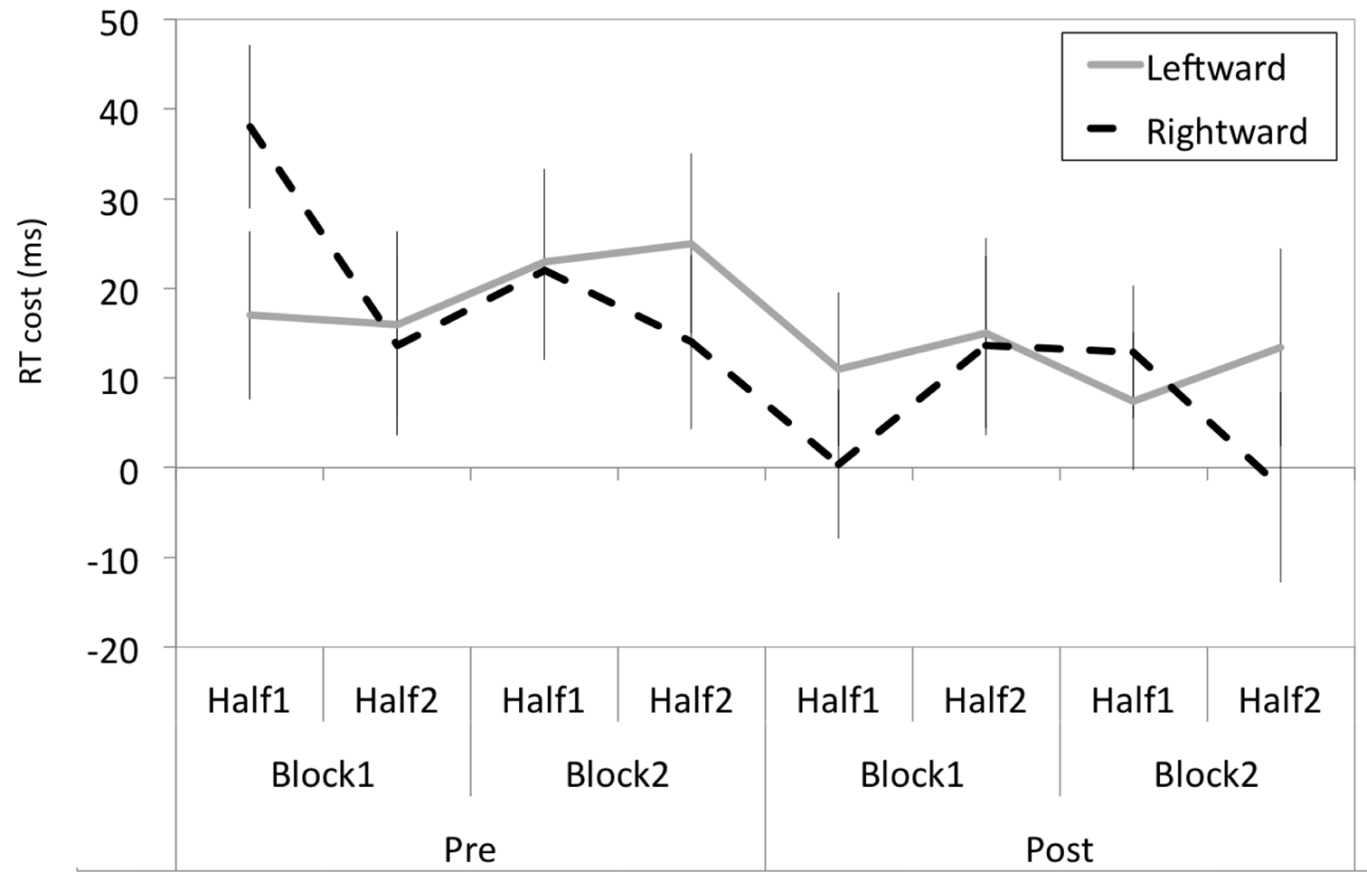
The RT costs of alignment for incongruent trials over time. The RT costs for the leftward- and rightward-shifting prism groups are shown broken into eight different time periods across the experiment, and were no different between the two groups.

There were no significant interactions of Prism and Congruency with any other factor with Block Number or Block Half to suggest any short-lived effect of prism adaptation on composite face processing. This is apparent in Figure 3, which shows incongruent trial RT costs of the two Prism groups averaged across eight time points (2 Prism x 2 Block Number x 2 Block Halves).

Overall, the results demonstrate that the RT cost of alignment became numerically smaller with time for both groups, consistent with a practice effect. Importantly, there was no significant reduction in RT cost following adaptation to leftward-shifting prisms.

## Discussion

Our results indicate that adaptation to leftward-shifting prisms did not reduce the RT cost associated with identifying individual halves of composite faces. Our data did reflect trends for reduced RT costs of alignment for incongruent faces for both the leftward- and rightward-shifting prism groups. However, this was not significant, and was in fact numerically larger for the rightward-shifting prism (control) group. With our large sample size (N=32 per group), it is unlikely that the lack of significant change in RT costs for incongruent trials can be attributed to type II error. We conclude instead that prism adaptation does not reduce configural processing of face stimuli.

Our research is particularly comparable to studies examining the effects of prism adaptation on the processing of chimeric faces and objects (stimuli that are formed by joining together the left and right halves of different faces or objects). Ferber and colleagues demonstrated that prism adaptation shifted the extent to which a neglect patient ^33^ and healthy participants ^34^ passed their gaze over different halves of chimeric faces. However, these changes in the visual exploration were not accompanied by any alteration in perceptual judgements of the faces. Sarri and colleagues ^35^ extended on this to demonstrate that although prism adaptation did not alter patients’ perception of chimeric faces it did dramatically improve their awareness of the identity of the left side of non-face objects. Our findings that prism adaptation alters the global versus local processing of Navon figures ^23, 24^ but not composite faces is consistent with this distinction between significant effects of prism adaptation on object but not face processing.

These results have bearing on an existing debate about whether the beneficial effects of prism adaptation on hemispatial neglect are restricted to tasks that have a direct motor or attentional component, or whether the technique also directly alters perceptual awareness per se ^33, 35– 39^. Striemer and Danckert ^36^ proposed that the beneficial effects of prism adaptation are limited to dorsal stream attentional and visuomotor behaviours, whereas ventral stream perceptual processes are relatively unaffected. Many of the tasks on which neglect patients have shown improvement following neglect, such as pen-and-paper tasks ^9, 40^, reading ^41^, haptic exploration ^12^, postural imbalance ^42^ and wheel-chair navigation ^43^, can be explained by a leftward shift in motor behaviour (including eye movements). In contrast, several studies have shown that prism adaptation does not alter the performance of neglect patients on tasks that require direct perceptual comparison of the left and right side of the stimuli ^33, 44, 45^, or stimuli on the left or right sides of space ^46^. Strikingly, the same patients showed leftward shifts in their ocular exploration of the stimuli ^33, 46^, or in similar tasks that had an overt motor component ^45^. Overall, Striemer and Danckert argued that prism adaptation alters performance on perceptual tasks only under specific circumstances (see Nijboer and colleagues ^47^ for data that directly contradicts this conclusion, and papers by Saevarsson and Streimer and their colleagues ^38, 39^ for further discussions of this model).

We previously attributed the effects of prism adaptation on the processing of Navon figures to changes in the relative activity of left and right temporo-parietal areas ^23, 24^. While object recognition per se is strongly attributed to dorsal stream processing, sensitivity to global versus local features of an object has been linked to differential specialisation of the left and right temporo-parietal cortices to these two levels of processing ^48– 53^. A further model of visual processing suggests that fast global processing of visual objects dominates in the dorsal stream providing rapid activation of frontoparietal attention mechanisms, whereas more detailed local processing occurs mainly through slower ventral stream mechanisms ^54– 56^. Thus, the effects of prism adaptation on the processing of Navon figures could be attributed to changes in dorsal stream mechanisms, either by altering relative processing weights of left and right temporo-parietal areas, or by a global enhancement or suppression of dorsal stream mechanisms.

Similar to other objects, it has been suggested that there is left hemisphere specialisation for processing face features and a right hemisphere specialisation for processing the face as a whole ^57^. However these have been localised to face-selective areas of the fusiform gyrus (i.e., the dorsal stream). A mechanism of prism adaptation that operates mainly through the ventral stream would therefore explain the absence of any effect of prism adaptation on face processing.

Prism adaptation is a promising treatment for hemispatial neglect. In order to understand the cognitive and neural mechanisms that underlie this intervention, it is important to examine tasks on which this technique has no impact, as well as those for which improvements are observed. Our finding that prism adaptation does not alter configural processing of faces is consistent with the dorsal versus ventral stream processing model proposed by Striemer and Danckert ^36^. Studies that directly compare the effects of prism adaptation on classic dorsal and ventral stream tasks would further illuminate the mechanisms of the beneficial effects of this intervention on hemispatial neglect.
