# Supplementary material for: Prism adaptation does not alter configural processing of faces
Source: F1000Res. 2013 Oct 14;2:215. [Version 1] doi: 10.12688/f1000research.2-215.v1 (PMC4111122; doi:10.12688/f1000research.2-215.v1)
Supplement: Peer review history

# Peer review: Referee response for version 1

**DOI:** 10.5256/f1000research.2468.r4653

**Published:** 2014-05-06

**Authors:** Wolfgang Heide

**Affiliations:** 1 Department of Neurology, General Hospital Celle, Celle, Germany

Bultitude and colleagues performed a psychophysical study on the perception/recognition time of composite faces before and after prism adaptation, in 64 healthy young women. Faces were composed of top and bottom halves from either the same or 2 different familiar persons. The authors demonstrate that adaptation to leftward-shifting prisms does not significantly change change the extent to which the irrelevant face half interfered with processing. Consequently, they conclude that prism adaptation does not have a significant effect on ventral stream processing of perceptual features, but appears to affect exclusively dorsal stream processing. The study results are straightforward and the discussion is appropriate. Only I propose that the authors might add some comments and discussion on their finding that rightward-shifting prisms did lead to some significant reduction of reaction time costs.

I have read this submission. I believe that I have an appropriate level of expertise to confirm that it is of an acceptable scientific standard.

# Peer review: Referee response for version 1

**DOI:** 10.5256/f1000research.2468.r3373

**Published:** 2014-02-18

**Authors:** Anna Barrett

**Affiliations:** 1 Translational Neurorehabilitation, Kessler Foundation Research Center, West Orange, NJ, USA

In this interesting study, Bultitude and colleagues demonstrate that prism adaptation does not alter the interference effect produced by irrelevant facial feature information on facial identification performance.  This is consistent with the findings of Sarri and colleagues ( 2006, 2011). The study results are straightforward and the discussion appropriate. With respect to theoretical implications of the results, there remains a question in my mind about whether this study supports a selective effect of prism adaptation on dorsal processing, versus a selective effect on spatial action-intentional “Aiming”, because previous studies demonstrated that face recognition with affectively-loaded, dynamic properties may be strongly linked to the dorsal visual stream ( Schwartz *et al*., 1998; Adolphs *et al.*, 2003). However, the current study did not use dynamic or (explicitly) emotional stimuli, so this distinction of dorsal visual processing versus spatial action processing is academic to the results at hand and the link of these results to dorsal versus spatial ‘Aiming’ function can be explored in future experiments.

I have read this submission. I believe that I have an appropriate level of expertise to confirm that it is of an acceptable scientific standard.

# Peer review: Referee response for version 1

**DOI:** 10.5256/f1000research.2468.r2070

**Published:** 2013-11-13

**Authors:** Christopher Striemer

**Affiliations:** 1 Department of Psychology, Grant MacEwan University, Edmonton, Canada

In this paper the authors have examined the after-effects of directional prism adaptation on global and local visual processing using the composite face effect. Previous work by Bultitude and colleagues has demonstrated that rightward prism adaptation in patients with right brain damage helps reduce the local processing bias. Furthermore, leftward prism adaptation in healthy individuals actually *increases* the local processing bias. Based on these findings, the authors predicted that, in healthy individuals, leftward prism adaptation should *increase* local processing, thereby *reducing* the composite face effect (i.e., the increase in reaction time observed when processing aligned vs. misaligned composite faces).

The results of the experiment indicated that there were no significant changes in the composite face effect following leftward prism adaptation. However, there was a trend towards a reduction in the composite face effect following rightward prism adaptation. Critically, the absence of any effect of leftward shifting prisms on the composite face effect cannot be attributed to de-adaptation, as participants remained significantly adapted at the conclusion of the experiment. Based on these results the authors argued that their data are consistent with the notion that prism adaptation primarily influences processing in the dorsal visual stream, and the dorsal attention network.

Overall I found the study to be very interesting and well motivated. Although I found study to be quite interesting, I do have some queries regarding the methods used, as well as the interpretation of the data.

In the Methods section it is not clear whether concurrent or terminal feedback was used during the prism adaptation session. Please clarify in the revised manuscript.

In the Results section, when discussing the results of the composite face task (page 5, 2 ^nd^ paragraph, right column) you note that, “the analyses revealed significant main effects of congruency for both leftward and rightward shifting prisms groups, reflecting lower RT costs of alignment for the incongruent faces than for the congruent faces.” Perhaps I have misinterpreted the composite face effect, but isn’t the prediction that participants should be slower (i.e., an *increased* RT cost) when processing aligned (compared misaligned) incongruent compared to congruent faces? The data from Figure 2 seem to support this interpretation in that participants are slower to respond for incongruent compared to congruent faces. Please clarify this in the revised manuscript.

It is interesting to note that the authors observed a trend towards a reduction in the composite face effect following rightward prism adaptation. However, the possible reasons for this are not addressed in the discussion. Is it possible that rightward prism adaptation may have increased activity in left temporal-parietal cortex thereby increasing attention to local features, and, by extension, decreasing configural face processing?

In the Discussion section I believe there may be a typo (or perhaps some confusion) regarding your characterization of the dorsal and ventral streams. Specifically, on the bottom of the left column on page 7 you mention that “While object recognition *per se* is strongly attributed to dorsal stream processing ....” I believe what you mean to say is that object recognition is strongly tied to the *ventral* visual stream. While it is true that some imaging studies have observed activation in dorsal stream areas during object processing tasks, it is as of yet unclear what visual information these signals are conveying. However, it is well known that damage to the ventral stream has devastating consequences for object and face recognition.

Likewise, when you are describing hemispheric specialization for face processing (page 7, middle paragraph, right column) you refer to the processing of face features and configural processing of faces as being “localized to face-selective areas in the fusiform gyrus (i.e., the dorsal stream).” Again, what I believe you meant to say was face selective areas in the *ventral* stream. 

Finally, your interpretation of the results is somewhat difficult to reconcile with findings from Sarri and colleagues ( Sarri, Greenwood, Kalra, & Driver, 2011; Sarri, Kalra, Greenwood, & Driver, 2006) suggesting that patients with neglect can detect chimeric non-face objects following rightward prism adaptation, as both types of patterns (i.e., chimeric objects and composite faces) require ventral stream processing. One way to interpret this is that perhaps prisms have differential effects on face vs. non-face objects. However, perhaps a simpler way of interpreting this is one of task difficulty. That is, discriminating between halves of a chimeric face, or the top and bottom halves of a composite face, require a detailed within-category discrimination. In contrast, chimeric objects typically involve a much simpler between-category discrimination.

I have read this submission. I believe that I have an appropriate level of expertise to confirm that it is of an acceptable scientific standard, however I have significant reservations, as outlined above.
